# Discharge against Medical Advice: A Case Study in a Public Teaching Hospital in Tehran, Iran in 2012

**DOI:** 10.5539/gjhs.v5n6p179

**Published:** 2013-09-27

**Authors:** Mohammadkarim Bahadori, Mehdi Raadabadi, Mohammad Salimi, Ramin Ravangard

**Affiliations:** 1 Health Management Research Center, Baqiyatallah University of Medical Sciences, Tehran, Iran; 2 School of Management and Medical Information, Kerman University of Medical Sciences, Kerman, Iran; 3 Student Scientific Research center, Tehran University of Medical Sciences, Tehran, Iran; 4 School of Allied Medical Sciences, Tehran University of Medical Sciences, Tehran, Iran; 5 School of Management and Medical Information Sciences, Shiraz University of Medical Sciences, Shiraz, Iran

**Keywords:** patient, hospital, discharge against medical advice (DAMA), Iran

## Abstract

Discharging against medical advice is to leave the hospital despite the advice of the doctor, which can result in complications and readmissions. This study aimed to examine the prevalence of patients’ discharge against medical advice (DAMA) and their reasons in a public teaching hospital in Tehran, Iran in 2012. This was an applied and cross-sectional study in which all patients (2601 patients) who had been discharged against medical advice from the studied hospital in 2012 were studied. Required data were collected using a data collection form. Collected data were analyzed using SPSS 18.0 and descriptive and analytical tests including Frequencies and Fisher’s Exact Test. The most and least common reasons for DAMA were, respectively, feeling complete recovery by patients (45.4%) and financial problems (1.3%). The results showed that there were significant differences between DAMA prevalence and patients’ sex and age (P<0.001). The prevalence of DAMA in the studied hospital was high and according to the existence of social work units in every hospital, it is recommended that patients’ consultation with the hospital social workers should be considered as an obligatory stage of the discharge against medical advice process in order to inform patients about its complications and adverse consequences.

## 1. Introduction

A medical team and the healthcare professionals during their careers are constantly faced with patients who, because of various reasons, refuse to receive care and want to leave hospital without receiving complete medical services. Such circumstances are inappropriate for both physicians and patients (Alfandre, 2009; Gunasekaran, Muthusamy, & Elder, 2013). Discharge against medical advice (DAMA) has emerged over the past three decades and is becoming one of the biggest preventable problems which forms approximately 2% of all hospital discharge (Ibrahim, Kwoh, & Krishnan, 2007).

However, why would DAMA is a problem? Results of many experimental studies have shown that patients discharged against medical advice have more readmission prevalence and also higher risk of complications than patients who receive care completely (Anis et al., 2002; Ding, Jung, Kirsch, Levy, & McCarthy, 2007; Hwang, Li, Gupta, Chien, & Martin, 2003; Valevski, et al., 2012). This increase in the readmission risk may be due to worsening of these patients’ conditions or because the patients have left care before achieving clinical stability (Southern, Nahvi, & Arnsten, 2012).

The results of Baptist and colleagues’ (2007) study showed that the probability of readmissions within the first 30 days after being discharged against medical advice for patients suffering from asthma was three times more than those who had received complete care. In some other studies, readmissions and even deaths have been reported as the complications caused by DAMA within less than 30 days of being discharged (Glasgow & Kaboli, 2010; Southern et al., 2012).

On the other hand, patients who are discharged against medical advice are known as a group threatened by the risks of mortality and infection. The results of a study showed that returning to the hospital to receive pharmaceutical care in the first 15 days after being discharged for patients who had been discharged against medical advice was up to 7 times more than other patients who had received complete care (Hwang et al., 2003). DAMA increases the probability of readmissions of the patients and, therefore, imposes additional costs on them. Aliyu in a study concluded that the costs imposed on patients because of being discharged against medical advice and readmissions due to that were 56% more than the costs of the initial hospitalization (Aliyu, 2002).

The results of some studies show that DAMA occurs in all types of diseases. The results of Fiscella and colleagues’ (2007) study showed that the risk of death for patients with acute myocardial infarction who had been discharged against medical advice was 60% more than those who had received complete care. DAMA in the US hospital emergency departments has become a major concern because approximately 500000 patients are annually discharged against medical advice, and about 1-2% of patients’ discharge from hospital emergency departments is DAMA which is increased to 6% for the hospitals located in deprived areas (Magauran, 2009).

There are several reasons for patients’ DAMA. Dissatisfaction with hospital care, lack of ability to pay hospital costs, family problems, lack of complete recovery, long stay in the hospital, feeling recovery, and location (urban or rural) are some of these reasons (Baptist et al., 2007). The results of a study on the causes of DAMA conducted in three major hospitals in Detroit during 1999-2005 showed that feeling recovery from the patients’ perspective, as well as, family problems were two main reasons for DAMA. The other important reasons were dissatisfaction with the medical team and the patient’s opinion of their competence and capability, etc. (Aliyu, 2002).

Results of many previous studies have shown that factors such as the presence of skillful and experienced physicians, not providing required special medical services due to lack of appropriate medical equipment and the overload of patients admitted to the hospital, and not paying full and close attention to the patients have effects on the prevalence of DAMA (Baptist et al., 2007). For example, the results of Chan and colleagues’ (2004) study showed that the probability of DAMA in HIV patients were higher than other patients because they received limited financial support. Patient’s inability to pay hospital costs is considered as an important factor in DAMA from the viewpoint of physicians (Wigder, Propp, Leslie, & Mathew, 2010). However, further studies should be conducted to prove this.

In Iran, there are several studies conducted on DAMA reasons. Rangraz Jeddi et al in their study concluded that patients’ personal problems, hospital staff, and hospital conditions were of the most important reasons for inpatients’ DAMA (Rangraz Jeddi, Rangraz jeddi, & Rezaeiimofrad, 2010). The results of Vahdat and colleagues’ (2012) study also showed that dissatisfaction with medical services, physicians’ suggestions, and dissatisfaction with the hospital facilities and equipment were the main reasons for inpatients’ DAMA.

According to what was mentioned above, it seems that health policy makers should pay more attention to DAMA and develop and implement some preventive strategies to deal with this problem. Considering that DAMA results in several problems for patients, this study aimed to examine the prevalence of DAMA and their reasons in a public teaching hospital in Tehran, Iran in 2012.

## 2. Methods

This was an applied and cross-sectional study conducted in a public teaching hospital in Tehran, Iran in 2012. This hospital was an educational, research and clinical center with 530 available beds, 30 units and departments, 30 polyclinics, 86.7% average bed occupancy rate, 7.23 days length of stay, and 50.17% bed turnover rate.

Among 18518 patients who had been discharged from the hospital during 2012, 2601 patients had been discharged against medical advice all of whom were studied. Required data were collected using a data collection form included four variables: age, sex, disease types, and the reasons for discharge against medical advice. The heads of the units asked the questions from the patients who wanted to discharge against medical advice and completed the form. Finally, the completed form should be signed by the patients or their relatives. The question about the reasons for discharging against medical advice was an open question and the patients could state any reason or reasons. Then, the reasons were categorized.

To collect the required data, the researchers referred to the Clinical Governance Unit of the hospital and received patients’ DAMA forms contained four mentioned variables. The disease types were determined by the units’ heads using patients’ charts and, then, categorized using International Classification of Diseases 10th Revision (ICD-10). Therefore, collected data were analyzed using SPSS 18.0 and descriptive and analytical tests including Frequencies and Fisher’s Exact Test. P<0.05 was considered statistically significant.

## 3. Results

The results showed that about 14% of inpatients had been DAMA during the studied period (given that of 18518 inpatients discharged, 2601 inpatients had been discharged against medical advice). It should be noted that there was not any readmission or discharging again against medical advice in the list of studied patients.

The disease types, as well as, the studied patients’ reasons for discharging against medical advice have been shown in Table 1. The most common disease types were, respectively, diseases of the circulatory system (30.8%), and injury, poisoning and certain other consequences of external causes (21.6%). On the other hand, the least common disease type was surgery and mental and behavioral disorders (each one 0.9%).

**Table 1:** The distribution of patients based on their disease types and their reasons for discharging against medical advice

	Reasons	Frequency(%)		Disease types	Frequency(%)
**Reasons for discharging against medical advice**	Feeling complete recovery	1181 (45.45)	**Patients’ disease types**	Diseases of the circulatory system	801 (30.8)
	Transferring to another hospital	393 (15.1)		Injury, poisoning and certain other consequences of external causes	561 (21.6)
	Lack of required facilities	191 (7.35)		Diseases of the nervous system	262 (10.1)
	The patient’s personal reasons	152 (5.8)		Diseases of the digestive system	249 (9.6)
	Not paying full and close attention to the patients	132 (5.1)		Diseases of the blood and blood-forming organs and certain disorders involving the immune mechanism	86 (3.3)
	Going to home	117 (4.5)		Diseases of the respiratory system	76 (2.9)
	Hospital intolerance	81 (3.1)		Diseases of the genitourinary system	66 (2.5)
	Prolonged treatment process	74 (2.85)		Symptoms, signs and abnormal clinical and laboratory findings, not elsewhere classified	56 (2.2)
	Dissatisfaction with the hospital	74 (2.85)		Mental and behavioral disorders	23 (0.9)
	Lack of required special medical services	43 (1.7)		Surgery	23 (0.9)
	Overcrowding in the unit and department	40 (1.5)		Others	258 (9.9)
	Financial problems	35 (1.3)		No Response	139 (5.3)
	Others	17 (0.7)		Total	2601 (100)
	No Response	71 (2.7)			
	Total	2601 (100)			

Also, 12 different main reasons were recognized for the patients’ DAMA of which the most and least common reasons for DAMA were, respectively, feeling complete recovery by patients (45.4%) and financial problems (1.3%) (Table 1).

Of 2601 patients who were discharged against medical advice in 2012, 65.3% were male and the majority of them (44.2%) were in the 19-39 age groups (Table 2). The results, also, showed that there was a significant difference in DAMA prevalence between the men and women (P<0.004) (Table 2). In other words, men were discharged against medical advice about 30% more than women. The difference in DAMA prevalence among different age groups was significant, too (P<0.000) (Table 2). The highest and lowest prevalence of discharging against medical advice were, respectively, in 19-39 and less than 19 age groups.

**Table 2:** The distribution of patients discharged against medical advice based on their sex and age

Reasons for discharging against medical advice	Sex (Frequency (%))			Age (Frequency (%))		

	Male	Female	Less than 19	19-39	40-59	More than 59
Not paying full and close attention to the patients	87 (3.4%)	45 (1.8%)	11 (0.4%)	48 (1.9%)	35 (1.4)	36 (5.3)
Feeling complete recovery	803 (31.8%)	377 (14.9%)	109 (4.4%)	591 (24%)	250 (10.1%)	211 (8.6%)
Overcrowding in the unit and department	23 (0.9%)	17 (0.7%)	4 (0.2%)	19 (0.8%)	7 (0.3%)	9 (0.4%)
Transferring to another hospital	252 (10%)	141 (5.6%)	32 (1.3%)	145 (5.9%)	98 (4%)	102 (4.1%)
Lack of required facilities	110 (4.3%)	81 (3.2%)	5 (0.2%)	50 (2%)	46 (1.9%)	84 (3.4%)
The patient’s personal reasons	104 (4.1%)	48 (1.9%)	11 (0.4%)	52 (2.1%)	36 (1.5%)	51 (2.1%)
Hospital intolerance	46 (1.8%)	35 (1.4%)	5 (0.2%)	32 (1.3%)	19 (0.8%)	21 (0.9%)
Going to home	76 (3%)	41 (1.6%)	12 (0.5%)	52 (2.1%)	18 (0.7%)	29 (1.2%)
Prolonged treatment process	54 (2.1%)	20 (0.8%)	1 (0.05%)	38 (1.5%)	14 (0.6%)	18 (0.7%)
Dissatisfaction with the hospital	40 (1.6%)	34 (1.3%)	2 (0.1%)	29 (1.2%)	19 (0.8%)	21 (0.9%)
Lack of required special medical services	23 (.0.9%)	20 (0.8%)	3 (0.1%)	14 (0.6%)	19 (0.8%)	6 (0.2%)
Financial problems	27 (1.1%)	8 (0.3%)	5 (0.2%)	15 (0.6%)	7 (0.3%)	7 (0.3%)
Others	7 (0.3%)	10 (0.4%)	1 (0.05%)	4 (0.2%)	7 (0.3%)	4 (0.2%)
Total	1652 (65.3%)	877 (34.7%)	201 (8.2%)	1089 (44.2%)	575 (23.3%)	599 (24.3%)
**P-value**	0.004			0.000		

## 4. Discussion

In every hospital and department, it can be seen patients who refuse the care provided and want to leave against medical advice which is potentially harmful for both doctor and patient, because the doctor may feel frustrated when s/he is unable to perform his/her job and duties, while the patient may threaten his/her life by leaving the hospital against medical advice and face with problems, such as readmission and health deterioration (Duñó, Pousa, Sans, Tolosa, & Ruiz, 2003).

This research aimed to study the prevalence of patients’ discharge against medical advice (DAMA) and their reasons in a public teaching hospital in Tehran, Iran in 2012.

The results of this study showed that about 14% of inpatients had been discharged against medical advice. Other studies have reported different prevalence of DAMA. In Iran, several studies have been conducted on DAMA. The prevalence of DAMA in the Manouchehri and colleagues’ (2012) study, conducted on the patients suffered from heart diseases and admitted to a cardiac hospital, was 4.9% and in the Roodpeyma’s (2010) study, conducted on the children admitted to the pediatric unit of a public hospital, was 5.3%. The prevalence of DAMA in Iran varied according to the types of diseases and hospitalization units. The results of Manouchehri and colleagues’ study and Roodpeyma’s study do not confirm the present study results because of the differences in their target populations. The present study was conducted in a public teaching hospital which its large numbers of hospital admissions was a reason for its high prevalence of DAMA, while the other mentioned studies were limited to a certain unit and certain patients.

The prevalence of DAMA in the Rangraz Jeddi and colleagues’ (2010) study and Kavosi and colleagues’ (2012) study, which had the target populations similar to the present study target population, were respectively, 10.3% and about 9% which were lower than our study prevalence. The reason for this difference can be attributed to the number of patients examined, so that the number of patients discharged against medical advice in the mentioned studies was lower than that in our study. In the Shirani and colleagues’ (2010) study, conducted in the emergency department of a teaching hospital, the prevalence of DAMA was 20.2%. This high prevalence of DAMA can be due to conducting this study in the emergency department.

In some studies conducted in Iran (Kavosi, et al., 2012; Rangraz Jeddi, et al., 2010; Vahdat, et al., 2012), the emergency department has been one of the hospital units which has the highest prevalence of DAMA. However, given that the prevalence of DAMA has not been studied based on the studied hospital units in the present study, the comparison between this study results and the results of Shirani and colleagues’ study is not possible, and conducting further researches is needed.

The prevalence of DAMA in public and teaching hospitals have been reported in several studies. For example, the prevalence of DAMA in the study of Weingart et al. (1998) conducted on an urban public teaching hospital was 0.8%, in the Southern and colleagues’ (2012) study was 2.4%, in the Schaefer and colleagues’ (2012) study conducted in a public hospital was 1% and in the Moyse and Osman’s (2004) study was 0.57%. The results of these studies indicate that there are differences between the prevalence of DAMA in Iran and other countries. The prevalence of DAMA are different among various diseases, too. Brook et al. (2006) studied 43 researches on psychiatric patients in their systematic review and showed that the range of DAMA prevalence was between 3% and 51% with the mean of 17%.

Several studies have examined the relationship between DAMA, age and sex. In the Manouchehri and colleagues’ (2012) study the prevalence of DAMA in men was higher than that in women. It can be probably due to studying the prevalence of DAMA in patients suffered from heart diseases in this study. Nasir and Babalola (2008) in their study found a significant relationship between sex and the prevalence of DAMA, so that the prevalence of DAMA in men was higher than that in women, which is consistent with the present study results. Also, there were significant relationships between age and the prevalence of DAMA in the Weingart and colleagues’ (1998) study, as well as, in the Ibrahim and colleagues’ (2007) study, so that the younger patients had higher prevalence of DAMA, which confirm the current study results.

In the present study, the studied patients discharged against medical advice (2601 patients) gave some reasons for their discharging against medical advice which the most common reason was feeling complete recovery by patients (1181 patients, 45.4%) and the least common reason was uncertainty of patients (only 1 patient, 0.03%). In a study conducted in Canada, from the 57 patients discharged against medical advice, 16 patients (28.07%) had been discharged because of family problems and 11 patients (19.3%) because of impatience (Hwang et al., 2003). The results of our study showed that 35 patients had been discharged against medical advice because of financial problems while the results of a study conducted in the US indicated that the main causes of discharging against medical advice were lack of health insurance coverage and low economic status and financial problems (Weingart et al., 1998) which are not consistent with the present study results.

This difference in these two study results may be due to the difference between target populations studied, so that in the present study only patients referred to a public and governmental hospital were studied. Usually the hospital costs in such hospitals are not too high and out of the patients’ ability to pay. Also, the difference in these two study results can be due to the special diseases treated in the studied hospital in the current study, so that their treatment was not so expensive. However, this issue was not reviewed in this study and needs further future studies. In the Schaefer and colleagues’ (2012) study, the most of medical staff believed that financial problems and the lack of appropriate insurance system were the main causes for discharging against medical advice while our results showed that only 1.3% of patients had been discharged against medical advice because of financial problems.

### 4.1 Study Limitations

There were some problems with using available DAMA forms to study the reasons for the patients’ DAMA including: failure to complete all items listed on the DAMA forms by patients, lack of some information such as the types of health insurance, education level, employment status, etc. Another limitation of our research was studying the patients discharged against medical advice only in one hospital.

## 5. Conclusions

The prevalence of DAMA in the studied hospital was high and had the significant relationships with patients’ sex and age. According to the high prevalence of DAMA in the studied hospital, as well as, the existence of social work units in every hospital, the prevalence of DAMA can be reduced by properly informing the patients admitted to the hospital and their relatives about DAMA adverse consequences. Also, considering the importance of patients’ complete recovery, and to avoid the complications of early discharge and discharge against medical advice, it is recommended that patients’ consultation with the hospital social workers should be considered as an obligatory stage of the discharge against medical advice process in order to inform patients about its complications and adverse consequences.
